# Characterization of Lipophilicity and Blood Partitioning of Pyrrolizidine Alkaloids and Their *N* -Oxides *In Vitro* and *In Silico* for Toxicokinetic Modeling

**DOI:** 10.1055/a-2523-3987

**Published:** 2025-02-21

**Authors:** Anja Lehmann, Manuel Haas, Julian Taenzer, Gerd Hamscher, Charlotte Kloft, Anja These, Christoph Hethey

**Affiliations:** 1 German Federal Institute for Risk Assessment (BfR), Berlin, Germany; 2 Department of Clinical Pharmacy and Biochemistry, Institute of Pharmacy, Freie Universitaet Berlin, Germany; 3 Institute of Food Chemistry and Food Biotechnology, Justus Liebig University, Giessen, Germany

**Keywords:** log *P*, octanol : water partition coefficient, blood-to-plasma ratio, drug distribution, pharmacokinetics, PBTK modeling

## Abstract

Lipophilicity and blood partitioning are important determinants for predicting toxicokinetics using physiologically-based toxicokinetic modeling. In this study, the logarithm of the *n* -octanol : water partition coefficient and the blood-to-plasma concentration ratio were, for the first time, experimentally determined for the pyrrolizidine alkaloids intermedine, lasiocarpine, monocrotaline, retrorsine, and their *N* -oxides. Validated *in vitro* assays for determination of the n-octanol : water partition coefficient (miniaturized shake-flask method) and the blood-to-plasma conentration ratio (LC-MS/MS-based depletion assay) were compared to an ensemble of *in silico* models. The experimentally determined octanol : water partition coefficient indicates a higher affinity of pyrrolizidine alkaloids and their *N* -oxides to the aqueous compared to the organic phase. Depending on the method, *in silico* determined n-octanol : water partition coefficients overpredicted the experimental values by ≥ 1 log unit for three out of four pyrrolizidine alkaloids (SPARC), four out of six (CLOGP), five out of eight (KowWIN), and three out of eight (S+log *P* ) pyrrolizidine alkaloids and their N-oxides. The blood-to-plasma concentration ratio obtained *in vitro* suggested a low binding affinity of pyrrolizidine alkaloids and their *N* -oxides towards red blood cells. For all eight pyrrolizidine alkaloids and their N-oxides, *in silico* predicted blood-to-plasma ratios deviated from experimental values by less than 50%. In conclusion, for physiologically-based toxicokinetic modeling of pyrrolizidine alkaloids and their *N* -oxides, the experimental octanol : water partition coefficient should be preferred, while the blood-to-plasma concentration ratio predicted by the acid/base classification model is a suitable surrogate for experimental data.

## Introduction

Pyrrolizidine alkaloids (PAs) are a large group of secondary plant metabolites of which the 1,2-unsaturated PAs are of particular concern due to their hepatotoxicity, genotoxicity, and potential carcinogenicity 1 . PAs and their corresponding *N* -oxides (PANOs) usually coexist in plants, while the latter are reduced to toxic PAs in both the intestine and liver 2 . The relative toxicity of structurally different PA congeners is substantially influenced by their individual kinetics, i.e., by their absorption, distribution, metabolism, and excretion (ADME) characteristics 3 . Physiologically-based toxicokinetic (PBTK) modeling is a powerful tool to explore the ADME characteristics of PAs within an organism. PBTK models have been successfully used for PAs to quantitatively predict *in vivo* liver toxicity and *in vivo* genotoxicity from *in vitro* toxicity data 4 , 5 . In conjunction with other physico- and biochemical properties, lipophilicity and blood partitioning are important determinants in PBTK models for predicting whole body toxicokinetics.

Since the *in vivo* analysis of tissue distribution requires animal testing that, apart from ethical concerns, is costly and time intensive, mechanistic models for an *a priori* prediction of tissue distribution have been established for PBTK modeling. In these mechanistic approaches, e.g., by Rodgers and Rowland 6 , 7 or by Schmitt 8 , 9 , the composition of the different tissues and the interaction of compounds with the tissue constituents is taken into consideration. For prediction of compound partitioning into the tissue lipids, a measure of lipophilicity is needed as a parameter in the models.

The *n* -octanol : water partition coefficient *K*_o : w_ ( *P* ) is a measure of lipophilicity in absence of relevant speciation and refers to the neutral form of a substance. *K*_o : w_ is defined as the concentration ratio in an organic phase, typically *n* -octanol, and an aqueous phase:

Ko:w = P= CorganicCaqueous{K}_{o:w}\ =\ P=\ {{C}_{organic}\over{{C}_{aqueous}}}

*K*_o : w_ is usually expressed as base 10 logarithm log *P* .

For ionizable substances, where neutral and charged species coexist to a relevant extent in solution, the corresponding distribution ratio *D* specifically accounts for neutral and ionized species 10 :

D= Cneutral,  organic + Cionized, organicCneutral,aqueous + Cionized, aqueousD=\ {{C}_{neutral,\ \ organic}\ {+}\ {C}_{ionized,\ organic}\over{{C}_{neutral,aqueous}\ {+}\ {C}_{ionized,\ aqueous}}}

Since the degree of ionization depends on pH, so does *D. D* is usually given as base 10 logarithm log *D.* The distribution coefficient log *D* can be calculated for a specific pH from log *P* and the acid dissociation constant p *K* a using the Henderson-Hasselbalch relation:

logD(pH)=logP - log(1 + 10(pH-pKa)Δi) logD(pH)=logP\ \hbox{‐}\ log(1\ {+}\ {10}^{(pH\hbox{‐}pKa)}\Delta i)

with Δi = 1 for acids and Δi = − 1 for bases 11 .

PAs are weak bases, since the nitrogen atom of the tertiary amine becomes protonated under neutral and acidic conditions. PANOs also show weak basic behavior, attributed to protonation of the oxygen atom of the *N* -oxide under acidic conditions. The weak basic characteristics of PAs and PANOs have been confirmed by *in silico* predicted p *K* a values (see **Table 1S** , Supporting Information).

Experimentally, the log *P* value can be determined using direct or indirect methods 12 . Direct experimental methods, such as the OECD shake flask method 13 or miniaturized adaptations 14 , obtain log *P* directly from the concentration ratio of the compound partitioned between the organic and aqueous phase. In indirect experimental methods, like reversed-phase HPLC (RP-HPLC) 15 , log *P* is estimated through the compound retention in a hydrophobic stationary phase. During experimental log *P* determination for ionizable substances, the impact of pH on the ionization state needs to be considered so that partitioning exclusively refers to their neutral form. In addition, a vast number of *in silico* methodologies have been developed to predict lipophilicity. While substructure-based models derive log *P* from cutting molecules into fragments or down to the single-atom level, property-based models use empirical approaches, the 3D structure of a molecule, or topological descriptors 16 . The lipophilicity of a compound is related to many biological properties, such as solubility and membrane permeability, thereby strongly affecting *in vivo* compound ADME characteristics and its pharmacodynamic and toxicological profile 17 .

Certain compounds are highly bound to or distributed into blood cells. Like plasma protein binding, this can significantly reduce a compoundʼs free plasma concentration, i.e., the fraction that is available for its pharmacological or toxicological action and its elimination. Blood partitioning, therefore, can strongly determine the toxicokinetic and toxicodynamic profile of a compound. The blood-to-plasma concentration ratio ( *R _b_* = *C*_blood_ / *C*_plasma_ ) is a measure of compound distribution within whole blood at equilibrium 18 .

The importance of a precisely determined *R _b_* value can be illustrated on, for example, the drugs thioridazine and maprotiline. For thioridazine, which shows an *R _b_* value of 0.55 19 and therefore almost no distribution into blood cells, exposure calculated from plasma equals twice the exposure calculated from whole blood. For maprotiline, which is distributed into blood cells, showing a *R _b_* value of 2.1 19 , exposure determined from plasma is half the exposure determined from whole blood. In the latter case, clearance calculated from plasma data would highly overestimate whole blood clearance if *R _b_* was not considered and could even exceed hepatic blood flow.

Experimentally, the *R _b_* value can be obtained *in vitro* by a separate measurement of compound concentrations in equilibrating plasma and whole blood (or blood cells) or, more laborious, *in vivo* within a pharmacokinetic study 20 . In addition, *in silico* methodologies for *R _b_* prediction have been developed. Some methodologies are based on mechanistic prediction of distribution into red blood cells 6 , 8 , while others comprise regression models that relate a variety of molecular descriptors to the *R _b_* value using statistical algorithms such as partial least squares regression and artificial neural networks 18 , 21 , 22 . In the absence of experimental data, it is often assumed *R _b_* = 1 for neutrals and bases, and *R _b_* = 1-hematocrit = 0.55 for acids and zwitterions (acid/base classification) 22 .

Due to the need to characterize lipophilicity and blood partitioning of PAs for PBTK modeling, the aim of this study was to determine log *P* and *R _b_* values of representative 1,2-unsaturated PA congeners. Therefore, PAs of different ester types were analyzed, including the monoester intermedine, the cyclic diesters monocrotaline and retrorsine, and the open-chained diester lasiocarpine. Additionally, the corresponding PANOs were included in the study. The log *P* and human *R _b_* values were determined *in vitro* using established experimental methods and compared to published literature values. The performance of established *in silico* models for the prediction of log *P* and *R _b_* values was assessed for PAs and PANOs. Log *P* values were predicted using two substructure- and two property-based models and *R _b_* values were predicted by an acid/base classification model and a mechanistic model. Finally, *in silico* predictions were evaluated against experimental values.

## Results

**Fig. 1:**
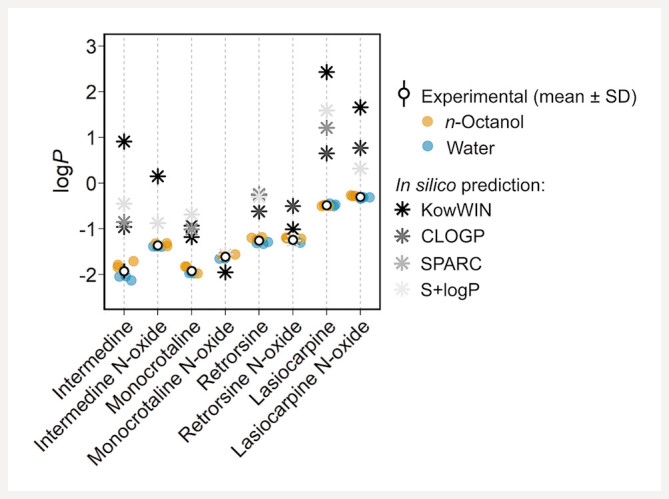
Experimentally determined and predicted log *P* values of PAs and PANOs. Experimental data are shown as the mean ± standard deviation (SD) of equilibration from the organic to the aqueous phase ( *n* = 3, yellow circles) and from the aqueous to the organic phase ( *n* = 3, blue circles). Log *P* predictions were not available for intermedine *N* -oxide, monocrotaline *N* -oxide (CLOGP), and for PANOs (SPARC).

Log *P* values were determined by bidirectional equilibration from *n* -octanol to water and vice versa (see section titiled Determination of *n* -octanol : water partition coefficient). Method evaluation with reference substances showed a convincing linear correlation (R ^2^ = 0.988) between determined log *P* values and literature log *P* values ( **Fig. 1Sa** , Supporting Information). Except for warfarin, determined log *P* values did not differ by more than 0.2 log units from those reported in literature demonstrating a high accuracy ( **Table 2S** , Supporting Information). The experimental method was precise, as indicated by relative standard deviations below 10% for sulfanilamide, tolbutamide, and warfarin (caffeine: 20%, theophylline: 47%).

For all PAs and PANOs, measured log *P* values were negative and ranged between − 1.93 (intermedine) and − 0.302 (lasiocarpine *N* -oxide) ( Table 1 and Fig. 1 , circles). There were no significant differences in log *P* values depending on the direction of equilibration, i.e., starting from the organic phase or starting from the aqueous phase. Log *P* values of PANOs were between 0.02 log units (retrorsine *N* -oxide) and 0.2 log units (lasiocarpine *N* -oxide), higher compared to log *P* values of the respective PAs.

**Table:** **Table 1** Experimentally determined and predicted log *P* values of PAs and PANOs.

Pyrrolizidine alkaloids and their *N* -oxides	Logarithm of the *n* -octanol:water partition coefficient (log *P* )					
	Experimental ^a^		*In silico* prediction			
			Substructure-based		Property-based	
			KowWIN 28	CLOGP 29	SPARC 30	S + log *P* 31
Intermedine	− 1.93	± 0.168	0.91	− 0.96	− 0.85	− 0.045
Intermedine *N* -oxide	− 1.36	± 0.0346	0.15	– ^b^	– ^b^	− 0.875
Lasiocarpine	− 0.487	± 0.0220	2.43	0.65	1.21	1.59
Lasiocarpine *N* -oxide	− 0.302	± 0.0269	1.66	0.77	– ^b^	0.319
Monocrotaline	− 1.92	± 0.0727	− 1.18	− 0.93	− 1.01	− 0.686
Monocrotaline *N* -oxide	− 1.61	± 0.0352	− 1.95	– ^b^	– ^b^	− 1.56
Retrorsine	− 1.26	± 0.0610	− 0.25	− 0.62	− 0.24	− 0.298
Retrorsine *N* -oxide	− 1.24	± 0.0421	− 1.01	− 0.50	– ^b^	− 1.26
^a^ Mean ± standard deviation of equilibration from the organic to the aqueous phase ( *n* = 3) and from the aqueous to the organic phase ( *n* = 3), ^b^ log *P* predictions not available						

The log *P* predicted by KowWIN deviated from experimental values by < 1 log unit for monocrotaline, monocrotaline *N* -oxide, and retrorsine *N* -oxide and by ≥ 1 log unit for the other PAs and PANOs ( Table 1 and Fig. 1 , asterisks). Log *P* calculated by the CLOGP method overpredicted experimental values by < 1 log unit for retrorsine and retrorsine *N* -oxide and by ≥ 1 log unit for the other PAs and PANOs. Regarding SPARC, log *P* values were overpredicted by < 1 log unit for monocrotaline and by ≥ 1 log unit for the other PAs. Note that log *P* predictions were not available for PANOs in SPARC. Log *P* predicted by the S + log *P* method (ADMET predictor) deviated from experimental values by < 1 log unit for all PANOs and retrorsine and by ≥ 1 log unit for intermedine, lasiocarpine, and monocrotaline.

**Fig. 2:**
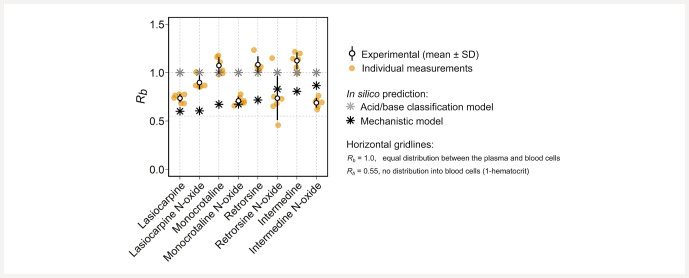
Experimentally determined and predicted *R _b_* values of PAs and PANOs. Experimental data are shown as the mean ± standard deviation (SD) ( *n* = 6).

The *R _b_* value was determined using the LC-MS/MS-based depletion assay that measures the compound concentration (i) in plasma that has been equilibrating with red blood cells and (ii) in a plasma reference (see section titled Determination of blood-to-plasma concentration ratio). The study was conducted in accordance with the Declaration of Helsinki and approved by the Ethics Committee of the Charité University Medicine Berlin (EA4/183/19, November 12, 2019). Method evaluation with reference substances showed a good linear correlation (R ^2^ = 0.908) between determined *R _b_* and literature *R _b_* ( **Fig. 1Sb** , Supporting Information). Except for imipramine and warfarin, the determined *R _b_* did not deviate by more than 10% from previously published *R _b_* values, which shows the high accuracy of the method ( **Table 2S** , Supporting Information). Relative standard deviations below 10% indicate that the experimental method was precise. An effect of the compound concentration on the *R _b_* value was not observed, within the range of 4 to 1000 ng/mL exemplarily determined for imipramine, caffeine, and warfarin ( **Fig. 2S** , Supporting Information).

**Fig. 3:**
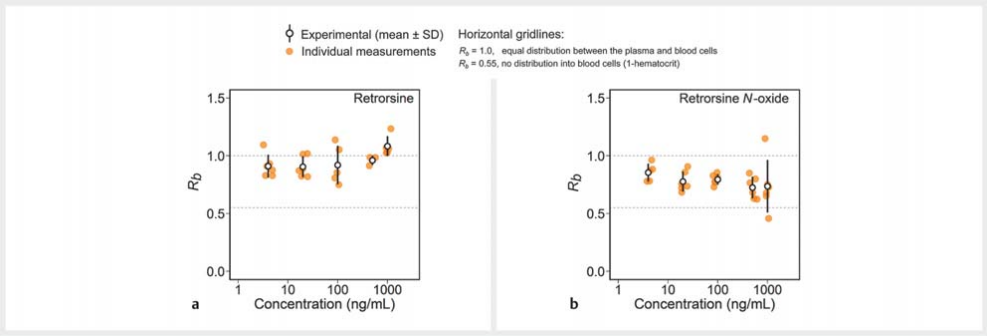
Effect of the concentration (4, 20, 100, 500, and 1000 ng/mL) on the *R _b_* value on the example of the PA retrorsine ( **a** ) and its *N* -oxide ( **b** ). Experimental data are shown as the mean ± standard deviation (SD), with *n* = 6 ( *n* = 3: retrorsine 500 ng/mL).

Measured *R _b_* values of PAs and PANOs ranged between 0.689 (intermedine *N* -oxide) and 1.12 (intermedine) ( Table 2 and Fig. 2 , circles). The *R _b_* value of the PAs intermedine, monocrotaline, and retrorsine was identical ( *R _b_* = 1.1). Except for lasiocarpine, determined *R _b_* values of PAs were 30 to 40% higher compared to that of their corresponding *N* -oxide. Within the range of 4 to 1000 ng/mL, no concentration dependency was observed, exemplarily determined for retrorsine and its *N* -oxide ( Fig. 3 a, b ).

**Table:** **Table 2** Experimentally determined and predicted *R _b_* values of PAs and PANOs.

Pyrrolizidine alkloids and their *N* -oxides	* Blood-to-plasma concentration ratio (R _b_* )			
	Experimental ^a^		*In silico* prediction	
			Acid/base classification model ^b^ (Eq. 6)	Mechanistic model (Eq. 7)
Intermedine	1.12	± 0.0853	1.00	0.807
Intermedine *N* -oxide	0.689	± 0.0499	1.00	0.867
Lasiocarpine	0.736	± 0.0438	1.00	0.601
Lasiocarpine *N* -oxide	0.897	± 0.0718	1.00	0.605
Monocrotaline	1.07	± 0.0865	1.00	0.672
Monocrotaline *N* -oxide	0.709	± 0.0430	1.00	0.674
Retrorsine	1.08	± 0.0859	1.00	0.717
Retrorsine *N* -oxide	0.736	± 0.228	1.00	0.830
^a^ Mean ± standard deviation ( *n* = 6), ^b^ at a physiological pH of 7.4, PAs are basic, while PANOs are neutral				

*In silico* predictions of *R _b_* according to the acid/base classification model deviated from experimentally determined *R _b_* by less than 10% for monocrotaline and retrorsine and by more than 10% but less than 50% for the other PAs and PANOs, showing a tendency for over prediction ( Table 2 and Fig. 2 , asterisks). Regarding the mechanistic model, *in silico* predictions of *R _b_* deviated from experimentally determined *R _b_* by less than 10% for monocrotaline *N* -oxide and by more than 10% but less than 50% for the other PAs and PANOs, with a tendency for under prediction.

## Discussion

We herein report, for the first time, lipophilicity and blood partitioning of representative PAs and their corresponding *N* -oxides, both being important predictors of compound ADME characteristics and key parameters in PBTK models. Measures of lipophilicity and blood partitioning, log *P* and *R _b_* values, were determined using published experimental assays as well as established *in silico* methods.

For all PAs and PANOs, log *P* values were negative, showing that the compounds have a higher affinity to the aqueous phase. The observed lipophilicity of analyzed PAs can be ranked as intermedine < monocrotaline < retrorsine < lasiocarpine. Regarding the PA ester type, these results suggest the following lipophilicity order, which is, however, limited by the small number of PAs assessed: monoester < cyclic diester < open-chained diester. In reversed-phase liquid chromatography, log *P* is correlated with the retention time, i.e., hydrophilic compounds elute first and lipophilic last 15 . Our observed lipophilicity ranking is supported by the elution order of 22 structurally different PAs in the reversed-phase chromatogram 23 . Monoesters and cyclic diesters tend to have shorter retention times, which implies that they have a lower lipophilicity compared to open-chained diesters. The experimental results indicate, except for lasiocarpine *N* -oxide, that PANOs are slightly more lipophilic compared to their respective PAs. This is in opposition to the *N* -oxidation of pyridines, aromatic weak bases with a chemical structure related to PAs, which was reported to increase hydrophilicity 24 .

Log *P* values calculated using *in silico* methodologies showed a trend for overprediction for PAs and PANOs. Predictions by substructure-based methods deviated upward from the experimental values by ≥ 1 log unit for five out of eight (63%) PAs and PANOs (KowWIN) and four out of six (67%) PAs and PANOs (CLOGP). Predictions by property-based methods deviated upwards by ≥ 1 log unit for three out of four (75%) PAs (SPARC) and three out of eight (38%) PAs and PANOs (S + log *P* ). The best performance was achieved with S + log *P* , which showed particularly good results for all PANOs, but not PAs.

For comparison, KowWIN, SPARC, and S + log *P* (CLOGP not available) showed better accuracy of log *P* for reference substances than for PAs and PANOs, i.e., deviations were < 1 log unit for five out of five (100%, KowWIN) and four out of five (80%, SPARC, S + log *P* ) reference substances (also see **Fig. 3S** , Supporting Information). The accuracy of log *P* prediction methods is strongly dependent on the compounds included in a training dataset and is limited by the complexity of molecules, in particular by their size 16 .

Exposure-related kinetic parameters, such as clearance, especially in relation to blood flow and volume of distribution, can be misleading if interpreted based on plasma instead of whole blood data, which can be prevented if the *R _b_* value of a compound is known. *R _b_* values below 1 ( *C*_plasma_ > *C*_blood_ ) indicate stronger binding to plasma proteins than to red blood cells, while *R _b_* values above 1 ( *C*_blood_ > *C*_plasma_ ) show that binding to blood cells is larger than plasma protein binding 18 . For PAs and PANOs, determined *R _b_* values were in the range between 0.689 and 1.12, with *R _b_* values of *N* -oxides tending to be smaller compared to PAs. These results suggest that PAs and PANOs have a low binding affinity towards red blood cells and distribution within red blood cells is not expected. This is in line with the absence of a concentration dependency of the *R _b_* value, as was exemplarily shown for retrorsine and its *N* -oxide. By contrast, for compounds that are highly bound or distributed into red blood cells, concentration-dependent saturation of binding or active uptake would have been expected 25 , 26 .

In the absence of experimental data, the *R _b_* value is often assumed to be 1 for neutrals and bases or 0.55 (1-hematocrit) for acids and zwitterions 22 . Since PAs are weak bases and PANOs are neutrals under physiological conditions, their *R _b_* value is 1 according to the acid/base classification model. While predictions by this acid/base classification model were slightly superior to predictions by the mechanistic tissue distribution model, both methods showed predictions close to experimental values, with deviations below 1.5-fold for all PAs and PANOs. In particular, *R _b_* values deviated from the experimental values by less than 10% for two out of eight (acid/base classification model) and one out of eight (mechanistic model) PAs and PANOs, and by more than 10% but less than 50% for six out of eight (acid/base classification model) and seven out of eight (mechanistic model) PAs and PANOs. It was reported that the performance of more complex artificial neural network (ANN) models was superior to that of mechanistic models for tissue distribution 18 , 22 . However, the application of ANN models is less feasible, since in addition to log *P* and fraction unbound in plasma ( *f*_u,p_ ), more compound-specific prior information (> 10 molecular descriptors) is required.

For PAs and PANOs, we recommend predicting tissue distribution with experimentally determined log *P* values, since the outlined deviations of *in silico* predictions from validated *in vitro* measurements were ≥ 1 log unit. A direct experimental method for log *P* determination, such as the miniaturized shake flask method used in this study, should be preferred over indirect methods to avoid erroneous results 27 . While *in silico* prediction of log *P* values was insufficient, the *in silico* predicted *R _b_* value using the acid/base classification model (deviations below 1.5-fold) can be regarded as a suitable surrogate for PAs and PANOs when experimental data on blood partitioning are not available.

## Material and Methods

### Materials

Intermedine (purity > 95%) and lasiocarpine (purity > 97%) were purchased from Phytoplan Diehm & Neuberger GmbH. Retrorsine (purity ≥ 90%) was obtained from Sigma-Aldrich Chemie GmbH. Monocrotaline (purity ≥ 90%), intermedine *N* -oxide (≥ 90%), monocrotaline *N* -oxide (≥ 90%), and retrorsine *N* -oxide (purity ≥ 90%) were received from Phytolab GmbH & Co. KG. Lasiocarpine *N* -oxide (purity 96.7%) was purchased from Cfm Oskar Tropitzsch GmbH. Caffeine (purity ≥ 99%), imipramine hydrochlorid (purity ≥ 99%), sulfanilamide (purity ≥ 99%), theophylline (purity ≥ 99%), tolbutamide (purity 99.8%), warfarin (purity ≥ 98%), and quinidine (purity ≥ 80%) were obtained from Sigma-Aldrich.

1-Octanol Chromasolv (purity ≥ 99%) was purchased from Fisher Scientific GmbH. Water LiChrosolv, acetonitrile LiChrosolv (purity ≥ 99.9%), and methanol LiChrosolv (purity ≥ 99.9%) were obtained from Merck KGaA. Ammonium formate Chromanorm (purity ≥ 99%) was provided by VWR International GmbH.

### Collection of whole blood and plasma samples

Blood samples were collected from two healthy donors (female, 44 years; male, 25 years). Collection of blood samples was conducted in accordance with the Declaration of Helsinki and was approved by the Ethics Committee of the Charité University Medicine Berlin (EA4/183/19, November 12, 2019). Whole blood was collected from the cubital vein into blood collection tubes with heparin (S-Monovette Lithium Heparin, Sarstedt AG & Co. KG). To obtain plasma, collection tubes were centrifuged for 10 min at 11 180 *g* and 8 °C and the plasma was separated from blood cells. Whole blood and plasma samples were stored at 4 °C until analysis.

### Determination of the *n* -octanol : water partition coefficient *P*

The *n* -octanol : water partition coefficient *P* was determined according to the miniaturized shake flask method 14 . Presaturated solutions of *n* -octanol and water were prepared. Therefore, equal volumes of *n* -octanol and water were mixed thoroughly in a separating funnel. After equilibration for 24 h, the organic and aqueous phases were separated for further use. The compounds were dissolved either in presaturated water or in presaturated *n* -octanol to measure bidirectional partitioning from the aqueous to the organic phase and vice versa. Next, 350 µL of compound solution were added to a reaction tube and the volume was made up to 500 µL with the same phase to achieve a concentration of 1000 ng/mL. Then, 500 µL of the other phase were added to obtain a total volume of 1 mL. Reaction tubes were shaken overnight at 500 rpm and 22 °C. After centrifugation for 20 min at 16 099 *g* and 20 °C, separated phases were removed using a glass syringe to avoid phase contamination. Of each sample, 200 µL were transferred to glass vials and analyzed by LC-MS/MS. Experiments were performed in triplicate for equilibration from the organic to the aqueous phase ( *n* = 3) and from the aqueous to the organic phase ( *n* = 3).

The log *P* values were calculated as logarithm of the mass-spectrometric peak area ratio (Eq. 4). Analyte concentrations were assumed proportional to peak areas since volumes of aqueous and organic phase were identical and samples were not diluted.

logP=logPeak area (Analyte in n-octanol)Peak area (Analyte in water)logP=log{Peak\ area\ (Analyte\ in\ n\hbox{‐}octanol)\over{Peak\ area\ (Analyte\ in\ water)}}

The experimental method was validated with the five structurally different reference substances caffeine, sulfanilamide, theophylline, tolbutamide, and warfarin. Linear correlation between determined and literature log *P* values was analyzed visually and numerically using the coefficient of determination R ^2^ . PAs and PANOs as well as reference compounds were neutral (≥ 96%) under experimental conditions (see **Table 1S** , Supporting Information).

The performance of *in silico* methodologies for prediction of the log *P* value was assessed against experimentally determined log *P* values for PAs and PANOs. To this end, two substructure-based prediction methods, KowWIN 28 and CLOGP (values published in 29 ), were compared to two property-based methods, SPARC 30 and S + log *P* (ADMET Predictor 31 ).

### Determination of blood-to-plasma concentration ratio

Conventionally, the partitioning of a compound between blood and plasma is determined by separate analysis of compound concentrations in equilibrating plasma and whole blood or red blood cells. Therefore, separate standards in their respective matrix, i.e., in plasma and whole blood or red blood cell lysates, are used. Here, we used an LC-MS/MS-based depletion assay. The blood-to-plasma concentration ratio *R _b_* was determined by measurement of compound concentrations in the plasma sample against a defined plasma reference 19 .

A whole blood sample and a plasma reference sample of the same volume were spiked with the compound to achieve a final concentration of 1000 ng/mL. Additional concentrations were assessed at 4, 20, 100, and 500 ng/mL to analyze concentration dependency of *R _b_* . The whole blood was incubated on a shaker for 60 min at 37 °C to allow equilibration of the compound between the plasma and red blood cells. Then, the whole blood was centrifuged for 10 min at 11 180 *g* and 8 °C to yield the plasma. The plasma reference was treated the same way as the whole blood regarding incubation and centrifugation. Then, 50 µL of plasma were collected from the separated whole blood and from the plasma reference, respectively. The collected plasma samples were quenched with 450 µL of ice-cold acetonitrile, mixed with a vortex mixer and centrifuged at 21 913 *g* for 10 min to remove plasma protein. Supernatants were collected and analyzed using LC-MS/MS. Experiments for PAs and PANOs were performed with *n* = 6 for PAs and PANOs and *n* = 4 for reference substances.

The *R _b_* value was calculated using the analyte peak area responses of the plasma samples (Eq. 5). Since volumes and matrix of the reference plasma sample and the equilibrating plasma sample were identical, analyte concentrations were proportional to the peak areas.

Rb=Peak area Analyte in plasma referencePeak area Analyte in equilibrating plasma{R}_{b}={Peak\ area\ \left(Analyte\ in\ plasma\ reference\right)\over{Peak\ area\ \left(Analyte\ in\ equilibrating\ plasma\right)}}

The experimental method was validated with the six structurally diverse reference substances, caffeine, imipramine, theophylline, tolbutamide, warfarin, and quinidine. The linear correlation between determined and literature *R _b_* values was assessed visually and numerically (R ^2^ ).

The performance of *in silico* models for the *R _b_* value was evaluated against the experimentally determined *R _b_* values for PAs and PANOs. To this end, an acid/base classification model and a mechanistic model for *R _b_* value prediction were used. In the acid/base classification model, the *R _b_* value was assumed 1 to be for neutrals and bases or 0.55 (1-hematocrit) for acids and zwitterions 22 :

Rb=1      for neutrals and bases0.55     for acids and zwitterions{R}_{b}=\left\{\matrix {{c}1\ \ \ \ \ \ for\ neutrals\ and\ bases\cr 0.55\ \ \ \ \ for\ acids\ and\ zwitterions}\right.

PAs and PANOs are weak bases (see **Table 1S** , Supporting Information, for p *K* a predictions). Under physiological conditions, i.e., at pH 7.4, PAs are present as bases, while PANOs are uncharged and therefore present as neutrals. Hence, according to the acid/base classification model, PAs and PANOs are assigned an *R _b_* value of 1.

In the mechanistic model, *R _b_* values are calculated from red blood cells to the unbound plasma partition coefficient *K*_rbc,pla_ by the Open Systems Pharmacology (OSP) Suite ( https://www.open-systems-pharmacology.org/ , accessed October 18, 2023) based on a mechanistic tissue distribution model. In this model, compound distribution into red blood cells is approximated by the distribution into the cellular constituents water, lipids, and proteins, instead:

Rb=(Krbc,pla× fu,p-1) ×hct-1{R}_{b}={(K}_{rbc,pla}\bullet \ {f}_{u,p}\hbox{‐}1)\ \bullet hct\hbox{‐}1

Krbc,pla=(frbc,water+ frbc,lipids×10logP+ frbc,prot×Kprot{K}_{rbc,pla}=({f}_{rbc,water}{+}\ {f}_{rbc,lipids}\bullet {10}^{logP}{+}\ {f}_{rbc,prot}\bullet {K}_{prot}

with *K*_rbc,pla_ = red blood cells to the unbound plasma partition coefficient; *f*_u,p_ = fraction unbound in plasma; hct = hematocrit; *f*_rbc,water_ = volume fraction of red blood cells that is water; *f*_rbc,lipids_ = volume fraction of red blood cells that is lipids; log *P* = logarithm to base 10 of the *n* -octanol : water partition coefficient; *f*_rbc,prot_ = volume fraction of red blood cells that is proteins; *K*_prot_ = water-protein partition coefficient.

Log *P* values were experimentally determined (see Table 1 ). The *f*_u,p_ values were taken from our in-house dataset (intermedine: 0.890, intermedine *N* -oxide: 1.08, retrorsine: 0.600, retrorsine *N* -oxide: 0.960; rapid equilibrium dialysis) or were predicted (lasiocarpine: 0.227, lasiocarpine *N* -oxide: 0.237, monocrotaline: 0.456, monocrotaline *N* -oxide: 0.461 32 ).

### Sample analysis by liquid chromatography and high-resolution mass spectrometry

The chromatographic measurements were performed using an UltiMate 3000 UHPLC system (Thermo Fisher Scientific) with a C18 Hypersil Gold column (150 mm × 2.1 mm; 1.9 µm particle size) and guard column (Thermo Fisher Scientific). The column temperature was set at 40 °C, while the flow rate and injection volume were adjusted to 0.3 mL/min and 2 µL, respectively. UHPLC was carried out by a binary mobile phase consisting of water (A) and methanol (B) as mobile phases, whereby 0.1% formic acid and 5 mM ammonium formate in each mobile phase was integrated. The gradient system was configured as follows: 0 – 7.0 min A: 95%, 7.0 – 7.5 min A: 50%, 7.5 – 7.6 min A: 20%, 7.6 – 10.1 min A: 0%, 10.1 – 15 min A: 95%.

High-resolution mass spectrometry was conducted using an Orbitrap mass spectrometer (Thermo Fisher Scientific) in the positive ionization mode (precursor ions [M + H] ^+^ ) to determine ion scans of reference substances caffeine, imipramine, sulfanilamide, theophylline, tolbutamide, quinidine, and warfarin. Ion scans (ddMS2) with collision energies in the range of 10 – 35 eV and a resolution of 17 500 were recorded, whereby three fragments

(product ions) with the highest relative abundances of each substance were identified ( **Table 3S** , Supporting Information). Representative ddMS2 data of the reference substances achieved with 25 eV are shown in **Figs. 4S-10S** , Supporting Information. For further analyses, samples containing reference substances were measured by data-independent acquisition (vDIA) consisting of full scans for quantification and MS2 data by fragmentation of four mass range windows ( *m/z* : 50 – 150, 130 – 300, 280 – 400, and 380 – 600) with a fixed collision energy of 25 eV for verification. Data were acquired and processed with TraceFinder 4.1 and Xcalibur 4.0 (Thermo Fisher Scientific).

Low-resolution mass spectrometry was carried out using an Agilent 6495 Triple Quadrupole system combined with an Agilent 1290 Infinity II LC System (Agilent Technologies) to measure PAs and PANOs. Compounds were ionized by electrospray ionization (ESI) in the positive mode and collision energies were individually adjusted. For identification, three product ions of each analyte were selected in each measurement ( **Table 3S** , Supporting Information). Data were acquired and processed with the Mass Hunter Workstation (Agilent Technologies).

Guidance 33 criteria for recovery (70 – 120%) and intraday precision (≤ 20%) were tested and fulfilled for reference substances in matrices water, *n* -octanol, and human plasma (also see **Table 4S** , Supporting Information).

### Data availability

All data are available in the GitHub repository https://github.com/al901 010/Supplement_Lipophilicity_BloodPartitoning_PAs .

## Contributorsʼ Statement

Conceptualization: A. Lehmann and C. Hethey; Methodology: A. Lehmann, M. Haas, A. These and C. Hethey; Validation: A. Lehmann, M. Haas, A. These and C. Hethey; Formal analysis: A. Lehmann and M. Haas; Investigation: M. Haas and J. Taenzer; Data curation: A. Lehmann; Writing – original draft: A. Lehmann; Writing – review & editing: M. Haas, J. Taenzer, G. Hamscher, C. Kloft, A. These and C. Hethey; Visualization: A. Lehmann; Supervision: G. Hamscher, C. Kloft, A. These and C. Hethey; Project administration: A. These

## Supporting Information

Linear regression of log *P* values ( **Fig. 1 aS** ), and *R _b_* values ( **Fig 1 bS** ) of reference substances experimentally determined in this work versus literature, the effect of the concentration on the *R _b_* value of reference substances ( **Fig. 2S** ), experimentally determined and predicted log *P* values of reference substances ( **Fig. 3S** ), representative ddMS2 data of reference substances ( **Figs. 4S** – **10S** ), predicted p *K* a values and predominant charge state at experimental pH (log *P* determination) of PAs, PANOs and reference substances ( **Table 1S** ), log *P* values and *R _b_* values of reference substances ( **Table 2S** ), MS/MS transitions and parameters ( **Table 3S** ), and mass spectrometric peak areas and calculated recovery of reference substances for log *P* determination ( **Table 4S** ) are available as Supporting Information.
Ergänzendes Material Supporting Information
