# Supplementary material for: Characterization of Lipophilicity and Blood Partitioning of Pyrrolizidine Alkaloids and Their N -Oxides In Vitro and In Silico for Toxicokinetic Modeling
Source: Planta Med. 2025 Feb 21;91(5):274–82. doi: 10.1055/a-2523-3987 (PMC12021451; doi:10.1055/a-2523-3987)
Supplement: Supplementary file 1 — Ergänzendes Material [file 10-1055-a-2523-3987-sup_pmi0497.pdf]

Supporting Information

Characterization of Lipophilicity and Blood Partitioning of Pyrrolizidine Alkaloids and Their *N*-Oxides *In Vitro* and *In Silico* for Toxicokinetic Modeling

Anja Lehmann[1, 2*], Manuel Haas[1*], Julian Taenzer[1], Gerd Hamscher[3], Charlotte Kloft[2], Anja These[1*], Christoph Hethey[1*]

[*]These authors contributed equally to this work.

Affiliations

[1]German Federal Institute for Risk Assessment (BfR), Berlin, Germany

[2]Department of Clinical Pharmacy and Biochemistry, Institute of Pharmacy, Freie Universitaet Berlin, Berlin, Germany

[3]Institute of Food Chemistry and Food Biotechnology, Justus Liebig University Giessen, Giessen, Germany

Correspondence

Anja Lehmann

Department of Clinical Pharmacy and Biochemistry

Institute of Pharmacy

Freie Universitaet Berlin

Kelchstr. 31

12169 Berlin

Germany

anja.lehmann@fu-berlin.de

Planta Med | DOI 10.1055/a-2523-3987 | © The Author(s).

Contents

Fig. 1S Linear regression of log*P* values (**a**) and R_b values (**b**) of reference substances experimentally determined in this work versus the literature.

Fig. 2S Effect of the concentration (4, 20, 100, 500, and 1000 ng/mL) on the R_b value of reference substances.

Fig. 3S Experimentally determined and predicted log*P* values of reference substances.

Fig. 4S ddMS2 spectrum of caffeine.

Fig. 5S ddMS2 spectrum of imipramine.

Fig. 6S ddMS2 spectrum of sulfanilamide.

Fig. 7S. ddMS2 spectrum of theophylline.

Fig. 8S. ddMS2 spectrum of tolbutamide.

Fig. 9S. ddMS2 spectrum of quinidine.

Fig. 10S. ddMS2 spectrum of warfarin.

Table 1S Predicted highest basic (strongest base) and lowest acidic (strongest acid) p*K*a values, as well as calculated charged species at experimental pH (log*P* determination).

Table 2S The log*P* values and R_b values of reference substances experimentally determined in this work versus the literature.

Table 3S MS/MS transitions and parameters.

Table 4S Mean peak areas (LC-MS/MS) and percentage recovery of log*P* determination.

Planta Med | DOI 10.1055/a-2523-3987 | © The Author(s).

Fig. 1S Linear regression of log*P* values (**a**) and R_b values (**b**) of reference substances experimentally determined in this work versus the literature. The red line represents the regression line. The inner dashed line represents the line of unity, while the outer lines in (**a**) indicate an error range of ± 1 log unit.

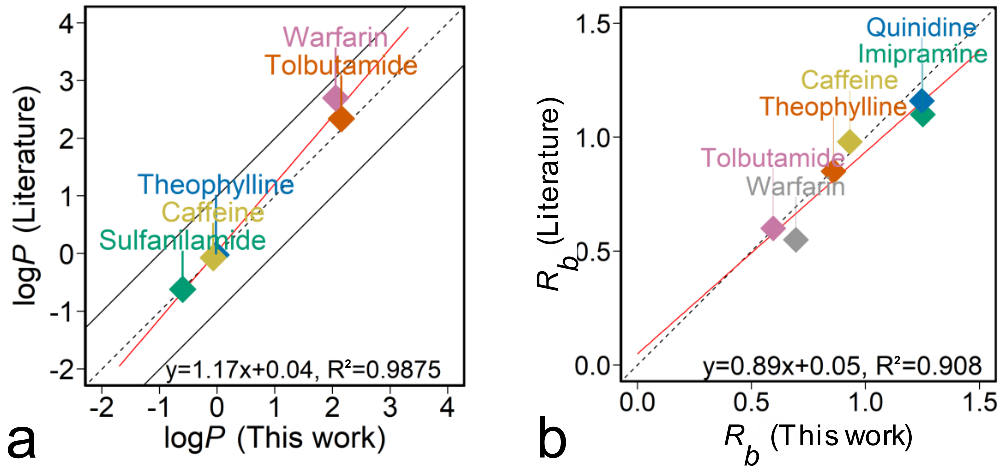

Fig. 2S Effect of the concentration (4, 20, 100, 500, and 1000 ng/mL) on the R_b value of reference substances. Experimental data are shown as the mean ± standard deviation (SD), with n = 3 (caffeine, imipramine) or n = 2 (warfarin).

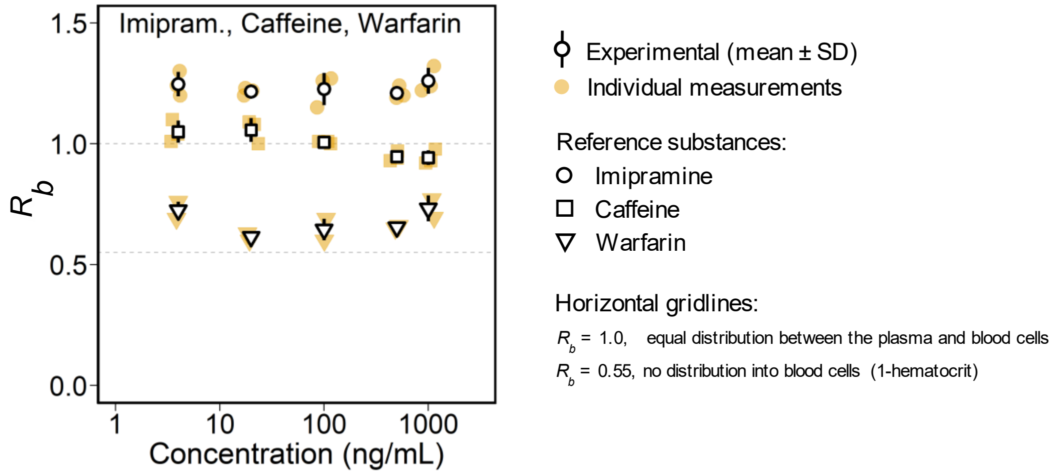

Planta Med | DOI 10.1055/a-2523-3987 | © The Author(s).

Fig. 3S Experimentally determined and predicted log*P* values of reference substances. Experimental data are shown as the mean ± standard deviation (SD) of equilibration from the organic to the aqueous phase (*n* = 3, yellow circles) and from the aqueous to the organic phase (*n* = 3, blue circles).

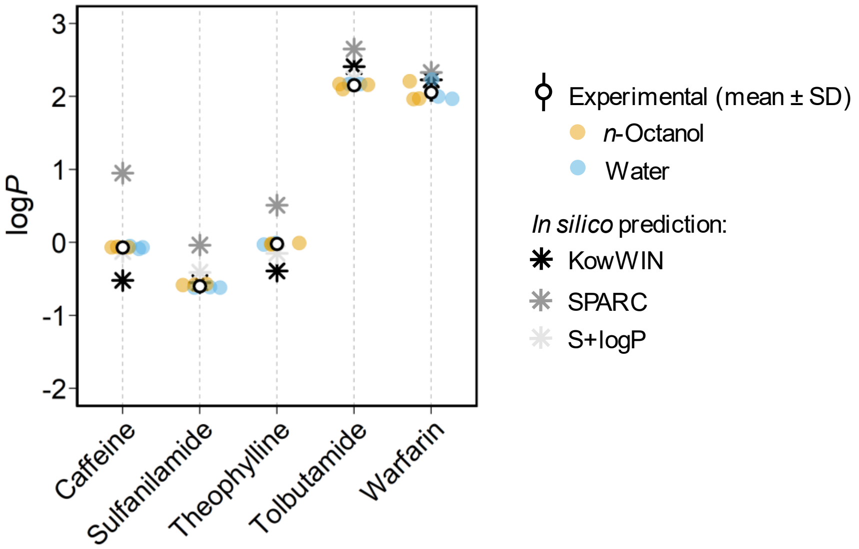

Fig. 4S ddMS2 spectrum of caffeine.

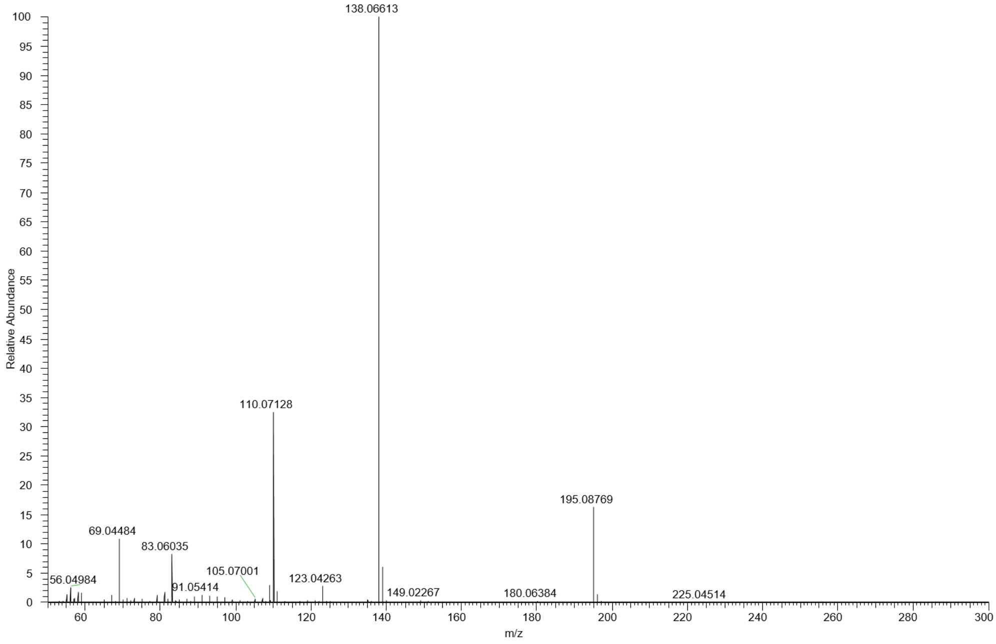

Planta Med | DOI 10.1055/a-2523-3987 | © The Author(s).

Fig. 5S ddMS2 spectrum of imipramine.

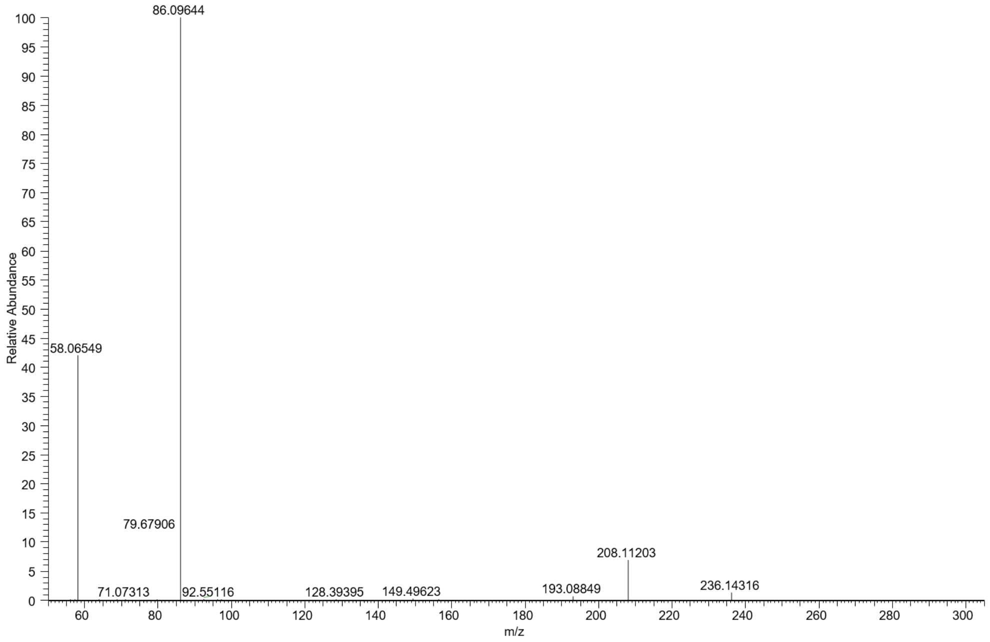

Fig. 6S ddMS2 spectrum of sulfanilamide.

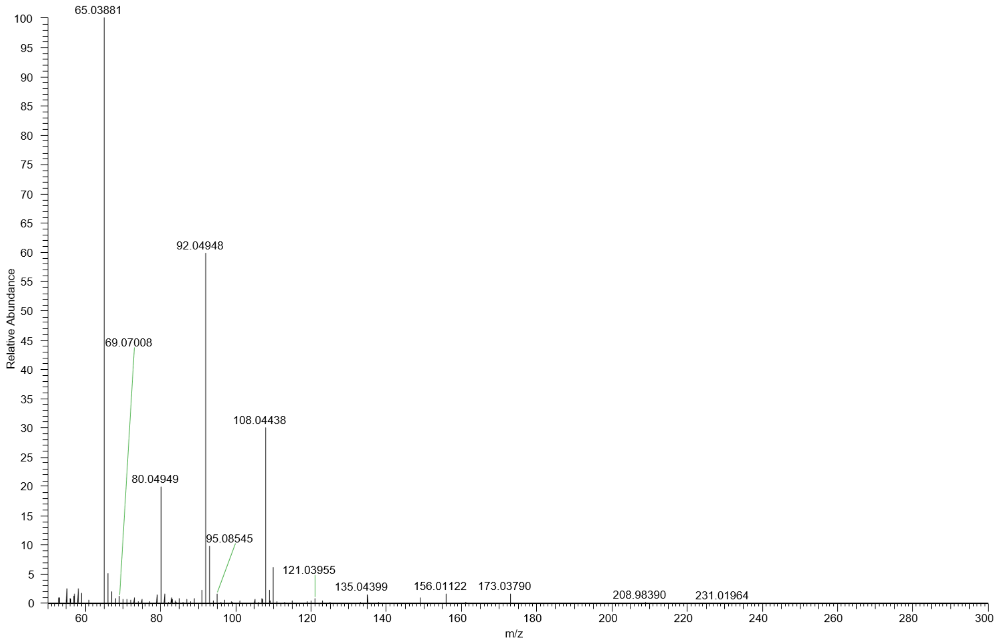

Planta Med | DOI 10.1055/a-2523-3987 | © The Author(s).

Fig. 7S ddMS2 spectrum of theophylline.

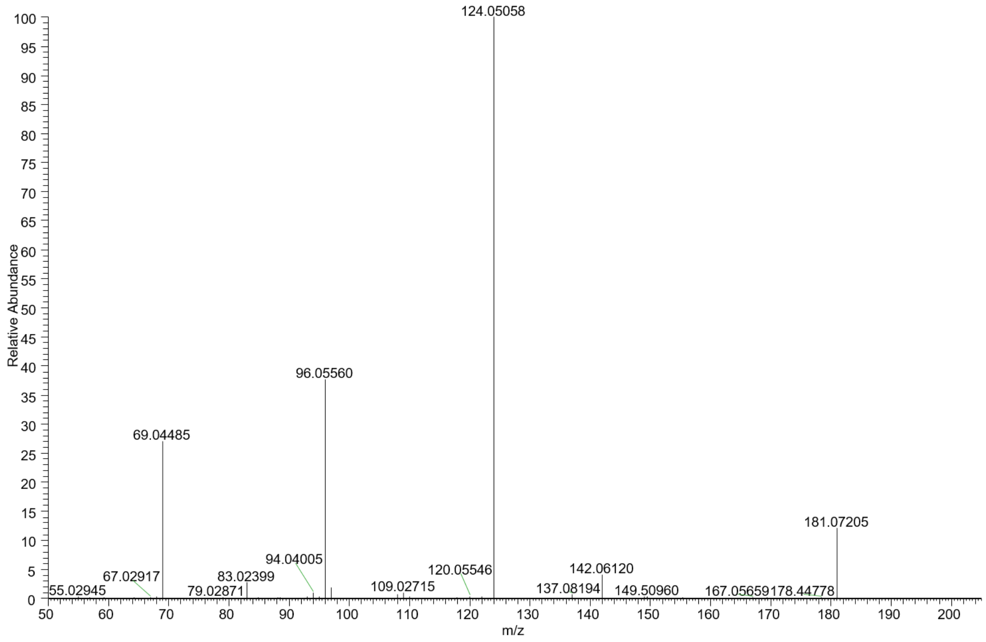

Fig. 8S ddMS2 spectrum of tolbutamide.

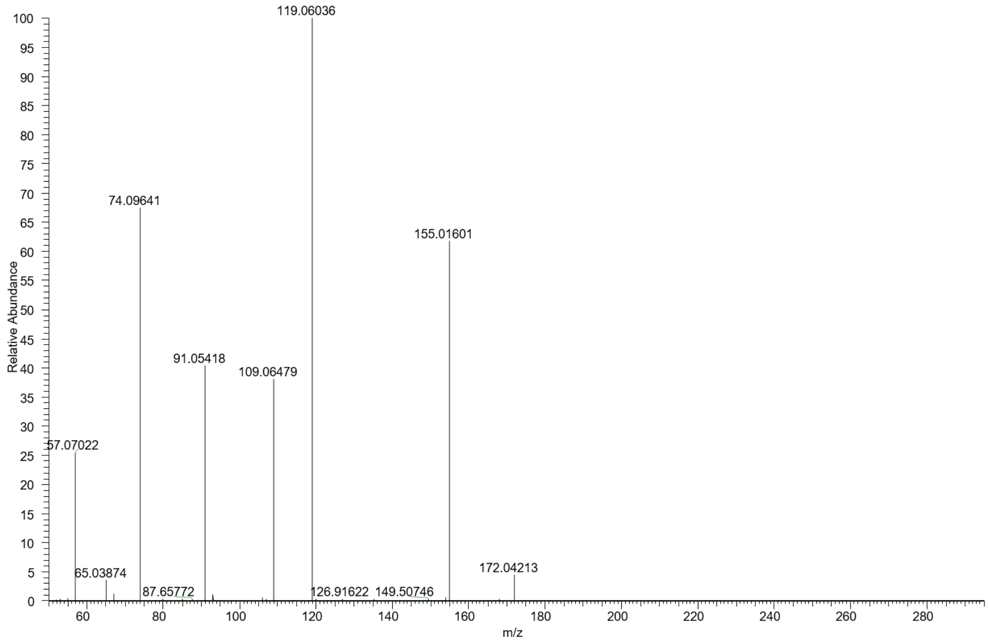

Planta Med | DOI 10.1055/a-2523-3987 | © The Author(s).

Fig. 9S ddMS2 spectrum of quinidine.

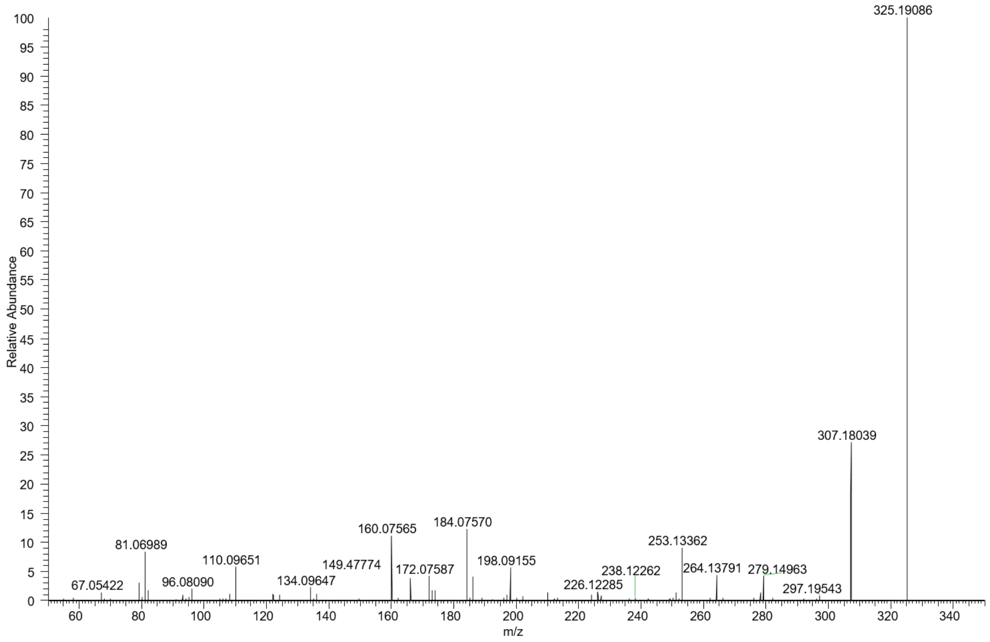

Fig. 10S ddMS2 spectrum of warfarin.

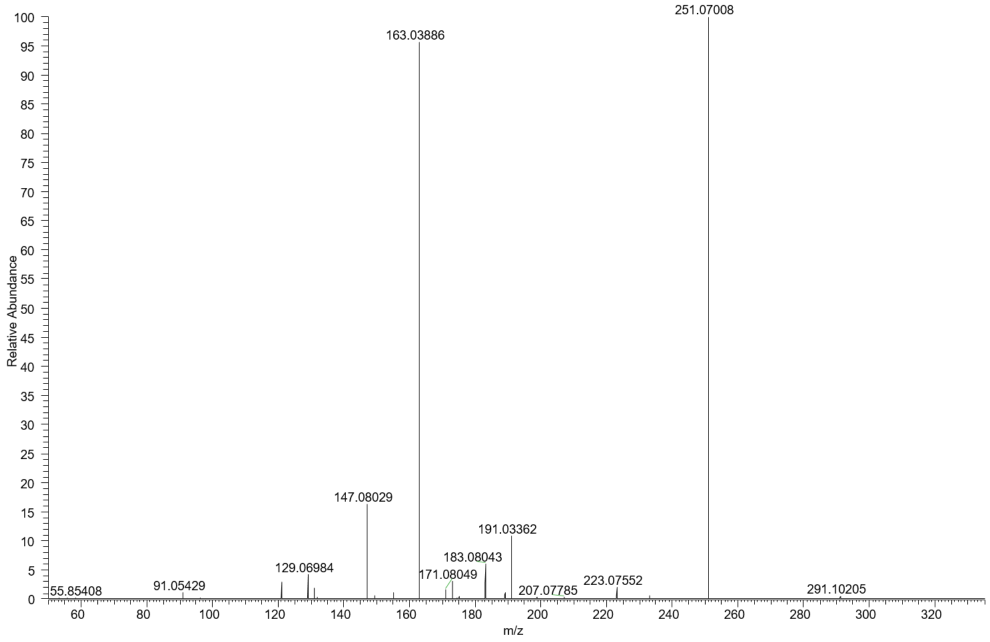

Planta Med | DOI 10.1055/a-2523-3987 | © The Author(s).

Table 1S Predicted highest basic (strongest base) and lowest acidic (strongest acid) p*K*a values, as well as calculated charged species at experimental pH (log*P* determination).

Compound	**Highest basic and lowest acidic p*K*a**[a]	**pH and predominant charge state at experimental conditions**	
	Base; acid	pH[b]	Charge state[c]
**Pyrrolizidine alkaloids and *N*-oxides**			
Intermedine	7.90; -	9	neutral (97%)
Intermedine *N*-oxide	4.67; -	6	neutral (97%)
Lasiocarpine	6.44; -	8	neutral (96%)
Lasiocarpine *N*-oxide	4.65; -	6	neutral (96%)
Monocrotaline	6.82; -	8	neutral (97%)
Monocrotaline *N*-oxide	4.60; -	6	neutral (97%)
Retrorsine	6.94; -	8	neutral (97%)
Retrorsine *N*-oxide	4.62; -	6	neutral (97%)
**Reference substances**			
Caffeine	1.43; -	3	neutral (98%)
Sulfanilamide	1.81; 10.6	8	neutral (98%)
Theophylline	1.32; 9.31	9	neutral (98%)
Tolbutamide	-; 5.21	4	neutral (98%)
Warfarin	-; 5.09	4	neutral (97%)

[a]p*K*a values predicted by ADMET Predictor [1]. [b]The experimental pH was calculated as pH = p*K*a + log($C_{\text{conjugate base}}$/$C_{\text{acid}}$) based on the nominal compound concentrations and autoprotolytic water activity with $C_{\text{conjugate base}}$ (µM) = 1 (ng/mL)/molar mass (g/mol) and C_{acid} (µM) = 0.1 for bases and vice versa for acids. the pH for amphoteric substances sulfanilamide and theophylline were calculated based on their strongest acid/base property. [c]The fraction neutral f_n was calulated as $f_n = 1/(1 + 10^{(pH - pKa)})$ for acids and $f_n = 1 - (1/(1 + 10^{(pH - pKa)}))$ for bases.

Table 2S The log*P* values and R_b values of reference substances experimentally determined in this work versus the literature.

Reference substance	**log*P***			R_b		
	**This work**[a]		**Literature**	**This work**[b]		**Literature**
Caffeine	-0.0683	± 0.0133	-0.07 [2]	0.931	± 0.0287	0.98 [3]
Imipramine	-		-	1.25	± 0.105	1.1 [4]
Quinidine	-		-	1.25	± 0.0971	0.92 [5], 1.4 [6]
Sulfanilamide	-0.598	± 0.0223	-0.62 [7]	-		-
Theophylline	-0.0197	± 0.00932	-0.02 [7]	0.860	± 0.00600	0.85 [8]
Tolbutamide	2.16	± 0.0274	2.34 [9]	0.595	± 0.0262	0.6 [10]
Warfarin	2.06	± 0.128	2.7 [11]	0.695	± 0.0436	0.55 [4]

[a]Mean ± standard deviation of equilibration from the organic to the aqueous phase (n = 3) and from the aqueous to the organic phase (n = 3). [b]Mean ± standard deviation with n = 4 (n = 3: caffeine; n = 2: warfarin).

Planta Med | DOI 10.1055/a-2523-3987 | © The Author(s).

Table 3S MS/MS transitions and parameters. QT: quantifier, QL: qualifier.

Compound	**Precursor ion (*m*/*z*)**	**Product ions (*m*/*z*) QT; QL; QL**	**Collision energy (eV) QT; QL; QL**
**Reference substances**			
Caffeine	195.1	138.1; 110.7; 108.7	15; 20; 10
Imipramine	281.2	86.1; 208.1; 193.1	15; 25; 35
Sulfanilamide	172.2	108.0; 156.0; 92.1	10; 10; 10
Theophylline	181.1	124.1; 142,1; 96.1	25; 25; 30
Tolbutamide	271.1	119.1; 155.0; 91.1	25; 20; 35
Quinidine	325.2	186.1; 307.2; 253.1	35; 35; 35
Warfarin	309.1	251.1; 163.0; 147.1	25; 15; 15
**Pyrrolizidine alkaloids and their *N*-oxides**			
Intermedine	300.2	120.1; 156.1; 138.1	26; 22; 30
Intermedine *N*-oxide	316.0	111.1; 172.1; 138.1	30; 30; 30
Lasiocarpine	412.2	120.1; 336.2; 220.1	18; 22; 30
Lasiocarpine *N*-oxide	428.2	136.1; 352.1; 254.1	38; 30; 26
Monocrotaline	326.2	120.1; 280.1; 194.1	40; 26; 30
Monocrotaline *N*-oxide	342.2	118.1; 138.1; 120.1	38; 30; 40
Retrorsine	352.2	120.2; 324.1; 138.2	34; 38; 30
Retrorsine *N*-oxide	368.2	120.1; 136.1; 118.1	38; 34; 40

Planta Med | DOI 10.1055/a-2523-3987 | © The Author(s).

Table 4S Mean peak areas (LC-MS/MS) and percentage recovery of log*P* determination measured with a sample size of $n = 3$ on three different days by two distinct analysts (a, b), accounting for the starting phase of equilibration (water, *n*-octanol). Relative dispersion reported as coefficient of variation (%CV). Intraday precision given as %CV of mean peak areas.

Reference substance	**Analyst**	**Starting phase of equilibration**	**Day 1**		**Day 2**		**Day 3**	
			Mean peak area (%CV)	%Recovery[a] (%CV)	Mean peak area (%CV)	%Recovery[a] (%CV)	Mean peak area (%CV)	%Recovery[a] (%CV)
Caffeine	a	water	$1.9{\cdot}10^5$ (1)	121 (0)	$2.7{\cdot}10^7$ (2)	114 (1)	$2.9{\cdot}10^7$ (2)	116 (1)
		n-octanol	$1.7{\cdot}10^5$ (1)		$2.3{\cdot}10^7$ (0)		$2.3{\cdot}10^7$ (1)	
	b	water	$1.9{\cdot}10^5$ (2)	115 (1)	$2.7{\cdot}10^7$ (4)	114 (2)	$2.8{\cdot}10^7$ (1)	113 (1)
		n-octanol	$1.7{\cdot}10^5$ (4)		$2.3{\cdot}10^7$ (2)		$2.4{\cdot}10^7$ (3)	
Sulfanilamide	a	water	$7.9{\cdot}10^7$ (1)	117 (2)	$7.0{\cdot}10^7$ (2)	104 (1)	$7.8{\cdot}10^7$ (2)	115 (2)
		n-octanol	$1.9{\cdot}10^7$ (3)		$1.7{\cdot}10^7$ (1)		$1.9{\cdot}10^7$ (2)	
	b	water	$7.4{\cdot}10^7$ (5)	111 (4)	$6.5{\cdot}10^7$ (1)	98 (2)	$7.2{\cdot}10^7$ (5)	107 (5)
		n-octanol	$1.9{\cdot}10^7$ (2)		$1.7{\cdot}10^7$ (2)		$1.9{\cdot}10^7$ (2)	
Theophylline	a	water	$4.9{\cdot}10^4$ (0)	92 (1)	$8.2{\cdot}10^6$ (1)	130 (1)	$8.5{\cdot}10^5$ (3)	103 (0)
		n-octanol	$4.5{\cdot}10^4$ (1)		$7.9{\cdot}10^6$ (2)		$8.5{\cdot}10^5$ (1)	
	b	water	$4.7{\cdot}10^4$ (2)	95 (2)	$8.0{\cdot}10^6$ (2)	120 (2)	$8.2{\cdot}10^5$ (2)	104 (2)
		n-octanol	$4.5{\cdot}10^4$ (2)		$7.9{\cdot}10^6$ (2)		$7.6{\cdot}10^5$ (1)	
Tolbutamide	a	water	$1.0{\cdot}10^5$ (2)	118 (2)	$3.8{\cdot}10^4$ (19)	108 (1)	$3.8{\cdot}10^4$ (12)	108 (1)
		n-octanol	$1.5{\cdot}10^7$ (2)		$5.6{\cdot}10^6$ (1)		$5.5{\cdot}10^6$ (1)	
	b	water	$9.9{\cdot}10^4$ (3)	114 (1)	$4.4{\cdot}10^4$ (13)	102 (1)	$3.6{\cdot}10^4$ (6)	95 (2)
		n-octanol	$1.5{\cdot}10^7$ (0)		$5.5{\cdot}10^6$ (1)		$5.2{\cdot}10^6$ (1)	
Warfarin	a	water	$2.2{\cdot}10^5$ (18)	85 (3)	$2.9{\cdot}10^7$ (10)	99 (2)	$3.5{\cdot}10^7$ (14)	106 (3)
		n-octanol	$2.1{\cdot}10^7$ (10)		$4.9{\cdot}10^9$ (2)		$3.2{\cdot}10^9$ (9)	
	b	water	$2.5{\cdot}10^5$ (3)	99 (1)	$3.2{\cdot}10^7$ (32)	99 (0)	$3.4{\cdot}10^7$ (3)	97 (2)
		n-octanol	$2.2{\cdot}10^7$ (1)		$5.0{\cdot}10^9$ (0)		$3.1{\cdot}10^9$ (2)	

[a]Recovery compares the total quantity of analyte present in both phases with the quantity of analyte originally introduced: %Recovery = 100% · (Peak area (Analyte in *n*-octanol) + Peak area (Analyte in water))/ Peak area (Analyte recovery sample).

Planta Med | DOI 10.1055/a-2523-3987 | © The Author(s).

References

[1] SimulationsPlus. ADMET Predictor 12. ADMET Property Estimation and Model Building, 2024

[2] Mirrlees MS, Moulton SJ, Murphy CT, Taylor PJ. Direct measurement of octanol-water partition coefficients by high-pressure liquid chromatography. J Med Chem 1976; 19: 615-619

[3] Gaohua L, Abduljalil K, Jamei M, Johnson TN, Rostami-Hodjegan A. A pregnancy physiologically based pharmacokinetic (p-PBPK) model for disposition of drugs metabolized by CYP1A2, CYP2D6 and CYP3A4. Br J Clin Pharmacol 2012; 74: 873-885

[4] Obach RS. Prediction of human clearance of twenty-nine drugs from hepatic microsomal intrinsic clearance data: An examination of *in vitro* half-life approach and nonspecific binding to microsomes. Drug Metab Dispos Biol Fate Chem 1999; 27: 1350-1359

[5] Sawada Y, Hanano M, Sugiyama Y, Iga T. Prediction of the disposition of nine weakly acidic and six weakly basic drugs in humans from pharmacokinetic parameters in rats. J Pharmacokinet Biopharm 1985; 13: 477-492

[6] Rodgers T, Leahy D, Rowland M. Physiologically based pharmacokinetic modeling 1: predicting the tissue distribution of moderate-to-strong bases. J Pharm Sci 2005; 94: 1259-1276

[7] Pyka A, Babuśka M, Zachariasz M. A comparison of theoretical methods of calculation of partition coefficients for selected drugs. Acta Pol Pharm 2006; 63: 159-167

[8] Ebden P, Banks J, Peel T, Buss DC, Routledge PA, Spragg BP. The disposition of theophylline in blood in chronic obstructive lung disease. Ther Drug Monit 1986; 8: 424-426

[9] Singh BN. A quantitative approach to probe the dependence and correlation of food-effect with aqueous solubility, dose/solubility ratio, and partition coefficient (Log *P*) for orally active drugs administered as immediate-release formulations. Drug Dev Res 2005; 65: 55-75

[10] Perkins EJ, Posada M, Kellie Turner P, Chappell J, Ng WT, Twelves C. Physiologically Based Pharmacokinetic Modelling of Cytochrome P450 2C9-Related Tolbutamide Drug Interactions with Sulfaphenazole and Tasisulam. Eur J Drug Metab Pharmacokinet 2018; 43: 355-367

[11] Hansch C, Leo A, Hoekman D. Exploring QSAR - Hydrophobic, Electronic, and Steric Constants. Washington, DC, USA: American Chemical Society; 1995: 161

Planta Med | DOI 10.1055/a-2523-3987 | © The Author(s).
